# Synergistic Antihypertensive Effect of *Carthamus tinctorius* L. Extract and Captopril in l-NAME-Induced Hypertensive Rats via Restoration of eNOS and AT_1_R Expression

**DOI:** 10.3390/nu8030122

**Published:** 2016-02-29

**Authors:** Putcharawipa Maneesai, Patoomporn Prasarttong, Sarawoot Bunbupha, Upa Kukongviriyapan, Veerapol Kukongviriyapan, Panot Tangsucharit, Parichat Prachaney, Poungrat Pakdeechote

**Affiliations:** 1 Department of Physiology, Faculty of Medicine, Khon Kaen University, Khon Kaen 40002, Thailand; pumaneesai@hotmail.com (P.M.); unicorn_fang-19@hotmail.com (P.P.); upa_ku@kku.ac.th (U.K.); 2 Faculty of Medical Sciences, Nakhonratchasima College, Nakhonratchasima 30000, Thailand; bugvo@hotmail.com; 3 Department of Pharmacology, Faculty of Medicine, Khon Kaen University, Khon Kaen 40002, Thailand; veerapol@kku.ac.th (V.K.); pantan@kku.ac.th (P.T.); 4 Department of Anatomy, Faculty of Medicine, Khon Kaen University, Khon Kaen 40002, Thailand; prapra@kku.ac.th

**Keywords:** *Carthamus tinctorius* L., captopril, l-NAME-induced hypertension, renin-angiotensin system, eNOS expression, oxidative stress

## Abstract

This study examined the effect of *Carthamus tinctorius* (CT) extract plus captopril treatment on blood pressure, vascular function, nitric oxide (NO) bioavailability, oxidative stress and renin-angiotensin system (RAS) in N^ω^-Nitro-l-arginine methyl ester (l-NAME)-induced hypertension. Rats were treated with l-NAME (40 mg/kg/day) for five weeks and given CT extract (75 or 150 or 300 or 500 mg/kg/day): captopril (5 mg/kg/day) or CT extract (300 mg/kg/day) plus captopril (5 mg/kg/day) for two consecutive weeks. CT extract reduced blood pressure dose-dependently, and the most effective dose was 300 mg/kg/day. l-NAME-induced hypertensive rats showed abnormalities including high blood pressure, high vascular resistance, impairment of acetylcholine-induced vasorelaxation in isolated aortic rings and mesenteric vascular beds, increased vascular superoxide production and plasma malondialdehyde levels, downregulation of eNOS, low level of plasma nitric oxide metabolites, upregulation of angiotensin II type 1 receptor and increased plasma angiotensin II. These abnormalities were alleviated by treatment with either CT extract or captopril. Combination treatment of CT extract and captopril normalized all the abnormalities found in hypertensive rats except endothelial dysfunction. These data indicate that there are synergistic antihypertensive effects of CT extract and captopril. These effects are likely mediated by their anti-oxidative properties and their inhibition of RAS.

## 1. Introduction

It is well established that nitric oxide (NO) is an endothelium-derived vasodilator and regulates vascular tone and blood pressure [1]. In the vasculature, NO is synthesized from the amino acid l-arginine using an endothelial nitric oxide synthase (eNOS) [2]. Chronic inhibition of continuous NO release by N^ω^-Nitro-l-arginine methyl ester (l-NAME), a nonselective nitric oxide synthase (NOS) inhibitor, produces systemic vasoconstriction and high blood pressure [3]. This is consistent with the reduction by l-NAME of plasma nitric oxide metabolites (NOx) and eNOS protein expression in vascular tissues [4]. l-NAME is also known to cause other cardiovascular abnormalities in rats, such as cardiovascular remodeling [4,5] and endothelial dysfunction [6]. Additionally, increased generation of reactive oxygen species (ROS) and decreased antioxidant defense systems have been observed in NO deficient rats [7]. Recent evidence shows in hypertension induced by NO inhibition, the increase in superoxide (O_2_^−^) production in vascular tissues and malondialdehyde (MDA) level in plasma is mediated by upregulation of nicotinamide adenine dinucleotide phosphate-oxidase (NADPH oxidase) subunit p47^phox^ [4]. Subsequently, several studies established that oxidative stress present in l-NAME hypertensive rats is associated with activation of RAS [8,9].

It is known that RAS plays a crucial role in development of hypertension, since angiotensin II (AngII) is a potent vasoconstrictor and can activate sympathetic nerve function. Ang II also causes heart and vascular remodeling and heart failure in models of hypertension [10]. There is substantial evidence that NO-deficient hypertension causes an imbalance of RAS in l-NAME hypertensive rats [11]. In l-NAME hypertensive rats, contractile response to AngII was enhanced via the action of Ang II type 1 receptor (AT_1_R). The authors suggest that this Ang II hypersensitivity in l-NAME-hypertensive rats maybe related to increased AT_1_R expression [12]. Further evidence for the important role of AT_1_R in l-NAME hypertensive rats is that fact that losartan, a selective AT_1_R antagonist, causes a greater fall in blood pressure (BP) in rats treated with l-NAME than in normotensive rats [13]. In addition, the Ang II effects are potentiated through enhanced ROS production via AT_1_R activation. Ang II-induced hypertensive animals show increased vascular O_2_^−^ production via membrane NADH/NADPH oxidase activation. This was confirmed by the observation that losartan abolishes the effect of Ang II-induced vascular O_2_^−^ production and endothelial dysfunction and hypertension [14].

Captopril, an angiotensin converting enzyme inhibitor, is a common drug for treatment of hypertension. In addition to inhibiting angiotensin II production, captopril also increases bradykinin levels and prostaglandin production, and exhibits antioxidant properties [15,16]. However, some complications of captopril treatment have been reported, including cough, loss of appetite, headache, and diarrhea [17]. Lowering the prescribed dosage of captopril may alleviate these side effects.

*Carthamus tinctorius* L. (CT) or safflower is widely used as a traditional Chinese medicinal herb to enhance blood circulation, alleviate blood stasis, and cerebrovascular and cardiovascular diseases [18,19]. There are increasingly extensive studies that demonstrate the biological activity of CT and its chemical constituents. It has been reported that CT contains many active compounds including quinochalones, flavonoids, alkaloids, polyacetylene, aromatic glucosides, and organic acids [19]. It is reported that hydroxyl safflor yellow A, an aquinochalone derivative, is the main active component of safflower [20]. Additionally, safflower has been shown to have anti-oxidative, anti-inflammatory, neuroprotective and anti-carcinogenic effects [19]. Han and coworkers demonstrated that safflower extract had a cardioprotective effect in myocardial ischemia induced by left anterior descending coronary artery occlusion in adult rats [21]. Liu and coworkers found that safflower yellow (SY) extracted from CT was able to reduce blood pressure, plasma renin activity, and Ang II levels in spontaneously hypertensive rats (SHR) [22]. However, there is little information regarding the antihypertensive effect of CT extract in l-NAME hypertensive rats. The aim of this study was to investigate whether CT extract or CT extract plus captopril could alleviate abnormalities in blood pressure, vascular function, NO inactivation, RAS, and oxidative stress in l-NAME-induced hypertensive rats.

## 2. Materials and Methods

### 2.1. Preparations of Carthamus Tinctorius (CT)

Dry CT was purchased from Vejpong Pharmacy, Co., Ltd. (Bankok, Thailand). Dry CT (1000 g) was soaked in 95% ethanol (2000 mL) for four hours. Thereafter, the ethanolic extract was filtered through nylon cloth and then dried using a spray dry machine. The yield (calculated using the dried powder extract) was 11.25% of the dry CT.

### 2.2. Animals and Experimental Protocols

Male Sprague-Dawley rats (220–240 g) were purchased from the National Laboratory Animal Center, Mahidol University, Salaya, Nakornpathom. Rats were maintained in an air-conditioned room (23 ± 2 °C) with a 12 h dark-light cycle at Northeast Laboratory Animal Center. All procedures complied with standards for the care and use of experimental animals and were approved by Animal Ethics Committee of Khon Kaen University, Khon Kaen, Thailand (AEKKU-NELAC 5/2557). After a one-week acclimatization period, the animals were divided into nine experimental groups, including two control groups that received tab water for five weeks and seven l-NAME treated groups that were treated with l-NAME (40 mg/kg/day) in their drinking water for five weeks (see Table 1, top row). Animals in the two control groups received either vehicle or CT extract (300 mg/kg/day) by intragastric administration for the last two weeks of the study. Animals in the seven l-NAME treated groups received l-NAME (40 mg/kg/day) and either vehicle or CT extract (75, 150, 300 or 500 mg/kg/day) or captopril (5 mg/kg/day) or a combination of CT extract (300 mg/kg/day) and captopril (5 mg/kg/day) by intragastric administration for the last two weeks of the study.

### 2.3. Indirect Measurement of Blood Pressure in Conscious Rats

Systolic blood pressure (SP) of rats was measured weekly using non-invasive tail cuff plethysmography (IITC/Life Science Instrument model 229 and model 179 amplifier; Woodland Hills, CA, USA) to assess blood pressure changes throughout the five weeks of the study.

### 2.4. Hemodynamic Measurement

At the end of the study, the animals were anesthetized by intraperitoneal injection of pentobarbital-sodium (60 mg/kg). A femoral artery was cannulated with a polyethylene tube. Thereafter, baseline values of SP, diastolic blood pressure (DP), mean arterial blood pressure (MAP), and heart rate (HR) were measured using the AcqKnowledge data acquisition and analysis software (Biopac Systems Inc., Santa Barbara, CA, USA). Hindlimb blood flow (HBF) was continuously measured by placing electromagnetic flow probes around the abdominal aorta connected to an electromagnetic flow meter (Carolina Medical Electronics, Carolina, NC, USA). Hindlimb vascular resistance (HVR) was calculated from baseline MAP and HBF (in 100 g tissue unit).

### 2.5. Isolated Aortic Rings Protocols

The thoracic aorta was rapidly removed and cut into rings 2–3 mm long for tension measurement. The tissue samples were mounted in a 15 mL bath containing Krebs’ solution at 37 °C and gassed with a 95% O_2_ and 5% CO_2_ gas mixture. Isometric contractions were recorded with a resting tension of 1 g using a transducer connected to a four-channel bridge amplifier and a PowerLab A/D converter and a PC running Chart v5 (PowerLab System, ADInstruments, NSW, Australia). The rings were allowed to equilibrate for 60 min before the first trial. The rings were pre-contracted with phenylephrine (10 µM), and then acetylcholine (Ach, 0.01–3 µM) or sodium nitroprusside (SNP, 0.01–3 µM) was added to the baths. The relaxation response was expressed as percent of the phenylephrine-induced contraction.

### 2.6. Perfusion Mesenteric Vascular Beds Protocols

After hemodynamic assessment, the animals were sacrificed by exsanguination. Mesenteric vascular beds were carefully isolated and then placed on a stainless steel grid (7 × 5 cm) in a humid chamber. The preparations were perfused with physiological Krebs’ solution at a constant flow rate of 5 mL/min for 60 min, using a peristaltic pump (07534-04, Cole-Palmer Instrument, Vernon Hills, IL, USA). Kreb’s solution is composed of the following (in mM units): NaCl 118, NaHCO_3_ 25, KCl 4.8, KH_2_PO_4_ 1.2, MgSO_4_·7H_2_O 1.2, CaCl_2_ 1.25 and glucose 11.1. The solution was maintained at 37 °C and continually gassed with a mixture of 95% O_2_ and 5% CO_2_ gas. Changes in perfusion pressure were recorded using the AcqKnowledge data acquisition and analysis software (Biopac Systems Inc.). The preparations were allowed to equilibrate for 30 min before the next trial. Thereafter, methoxamine (5–7 µM) was added into Kreb’s solution to raise tone (70–90 mmHg above baseline). To determine vasoactive performance of resistance small arteries, different doses of vasoactive agents, Ach (1 nM–0.01 µM) or SNP (1 nM–0.01 µM), were injected through neoprene rubber tubing proximal to the tissue.

### 2.7. Assay of Vascular O_2_^−^ Production

Productions of O_2_^−^ in vascular tissues were determined by lucigenin-enhanced chemiluminescence as previously described [23] with some modification. The carotid artery was rapidly removed and placed in ice-cold saline, and connective tissues and adherent fat was cleaned off. The vessel was cut into 1 cm lengths and incubated with 1 mL oxygenated Krebs-KCl buffer and allowed to equilibrate at pH 7.4, 37 °C for 30 min. Thereafter, lucigenin was added to the sample tube and placed in a luminometer (Turner Biosystems, Sunnyvale, CA, USA). Luminometer counts were recorded every 30 s for 5 min and averaged. Vascular tissue O_2_^−^ production was expressed as relative light unit count per minute per milligram of a dried tissue weight.

### 2.8. Assay of Plasma Malondialdehyde (MDA)

The concentration of plasma MDA was measured as TBA reactive substances by a spectrophotometric method, as previously described [24]. In brief, 150 μL plasma samples were reacted with 10% TCA, 5 mmol/L EDTA, 8% SDS, and 0.5 μg/mL BHT. The mixture was incubated for 10 min at room temperature, then 0.6% TBA was added and the mixture was boiled in a water bath for 30 min. After cooling to room temperature, the mixture was centrifuged at 10,000 g for 5 min. The absorbance of the supernatant was measured at 532 nm by a spectrophotometer (Amersham Bioscience, Arlington, MA, USA). A standard curve was generated at different concentrations from 0.3 to 10 μmol/L using 1,1,3,3-tetraethoxypropane.

### 2.9. Assay of Plasma Ang II and Plasma Nitric Oxide Metabolites (NOx)

The concentration of plasma Ang II was measured using an angiotensin II EIA kit (St. Louis, MO, USA). Plasma NOx was assayed using an enzymatic conversion method [25] with some modifications [26]. In brief, plasma sample were deproteinized by ultrafiltration using centrifugal concentrators (Pall Crop., Ann Arbor, MI, USA). The supernatant was mixed with 1.2 µmol/L NADPH, 4 mmol/L glucose-6-phosphate disodium, 1.28 unit/mLglucose-6-phosphate dehydrogenase, and 0.2 U/mL nitrate reductase, and then incubated at 30 °C for 30 min. The mixture was then reacted with Griess solution (4% sulfanilamide in 0.3% NED) for 15 min. The absorbance of samples at a wavelength of 540 nm was measured on a microplate reader (Tecan GmbH., Grödig, Austria).

### 2.10. Western Blot Analysis

Protein eNOS and AT_1_R expression levels were determined in aortic tissue homogenates following a previously described Western blot method [27] with some modifications. Homogenates were electrophoresed on a sodium dodecyl sulfate polyacrylamide gel electrophoresis system. The proteins were electrotransferred onto a polyvinylidenedifluoride (PVDF) membrane and blocked with 5% skimmed milk in phosphate buffer saline with 0.1% Tween-20 (PBST) for two hours at room temperature before overnight incubation at 4 °C with mouse monoclonal antibodies to eNOS (BD Biosciences, San Jose, CA, USA), rabbit polyclonal antibodies to AT_1_R (Santa Cruz Biotechnology, Indian Gulch, CA, USA) and goat polyclonal IgG to β-actin (Santa Cruz Biotechnology). The membranes were washed with PBST and then incubated for two hours at room temperature with horseradish peroxidase conjugated secondary antibody. The blots were developed in Amersham™ ECL™ Prime solution (Amersham Biosciences Corp., Piscataway, NJ, USA) and densitometric analysis was performed using an ImageQuant™400 imager (GE Healthcare Life Science, Piscataway, NJ, USA). The intensity of the bands was normalized to that of β-actin and data were expressed as a percentage of the values determined in the control group from the same gel.

### 2.11. Statistical Analysis

Data are expressed as mean ± S.E.M. The differences among treatment groups were analyzed by one-way analysis of variance (ANOVA) followed by post-hoc Student Newman-Keul’s test. A *p*-value of less than 0.05 was considered statistically significant.

## 3. Results

### 3.1. Effects of CT Extract and Captopril Supplementation on Systolic Blood Pressure in Conscious Rats

**Figure 1:**
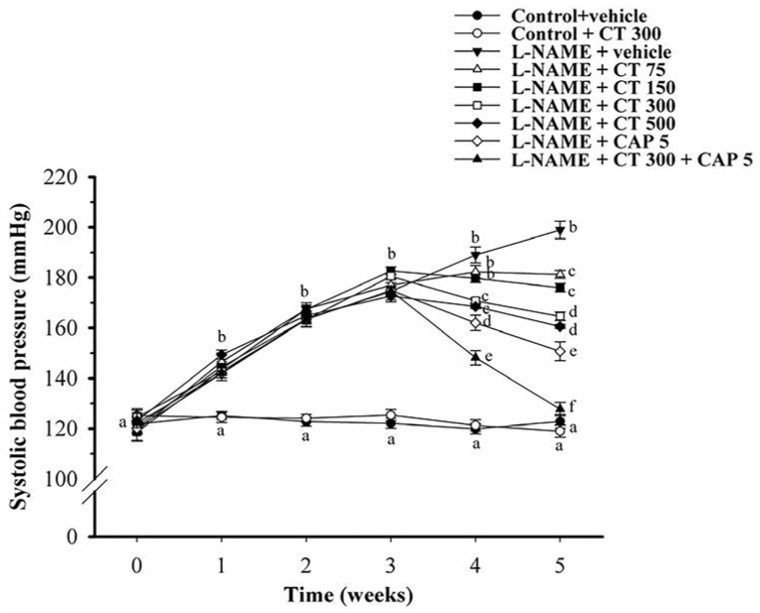
Effects of CT extract and captopril supplementation on systolic blood pressure in all groups. Data are expressed as mean ± SEM (*n* = 12 in each group). Data bearing different letters were significantly different (*p* < 0.05).

At baseline, there was no significant difference in SP among experimental groups. Daily administration of l-NAME for five weeks caused significant increase in SP (198.9 ± 3.2 mmHg), comparing to those of control group (122.8 ± 2.8 mmHg) (*p* < 0.05). Treatment with CT extract (75, 150, 300 and 500 mg/kg/day) for two weeks significantly reduced SP in a dose-response manner (181.2 ± 1.5, 176 ± 1.7, 164 ± 1.4 and 157.9 ± 1.3 mmHg, respectively). However, there was no significant difference in SP between dose 300 mg/kg/day and 500 mg/kg/day of CT extract. Therefore, CT extract at dose 300 mg/kg/day is the optimal dose to treat hypertension in this animal model. CT extract (300 mg/kg/day) had no effect on SP in normal control rats (119 ± 2.4 mmHg) (*p* < 0.05). Treatment with captopril (5 mg/kg/day) for the last two weeks significantly decreased SP in hypertensive rats (150.71 ± 3.24 mmHg) compared to the untreated rats (*p* < 0.05). l-NAME hypertensive rats treated with CT extract (300 mg/kg/day) plus captopril (5 mg/kg/day) restored SP back to the control level (127.74 ± 2.54 mmHg; *p* < 0.05) (Figure 1).

### 3.2. Effects of CT Extract and Captopril Supplementation on Hemodynamic Parameters

After the five week study period, a marked increase in SP, DP, MAP and HR was found in l-NAME hypertensive rats compared to control rats (*p* < 0.05). Moreover, increases in HVR and decreases in HBF were also observed. Treatment with CT extract (75, 150, 300 and 500 mg/kg/day) caused a significant decrease in SP, DP, MAP and HR in a dose-dependent manner compared to the untreated group (*p* < 0.05). Improvement of HBF and HVR was observed at a dose of 300 and 500 mg/kg/day compared to untreated group (*p* < 0.05). In addition, CT extract did not affect hemodynamic status in control rats (Table 1). Captopril also improved all hemodynamic parameters compared to untreated hypertensive rats (*p* < 0.05). Interestingly, combination treatment of CT extract and captopril restored hemodynamic parameters in hypertensive rats back to the control level (Table 1).

### 3.3. Effects of CT Extract and Captopril Supplementation on Vascular Reactivity in Aortic Rings and Mesenteric Vascular Beds

**Figure 2:**
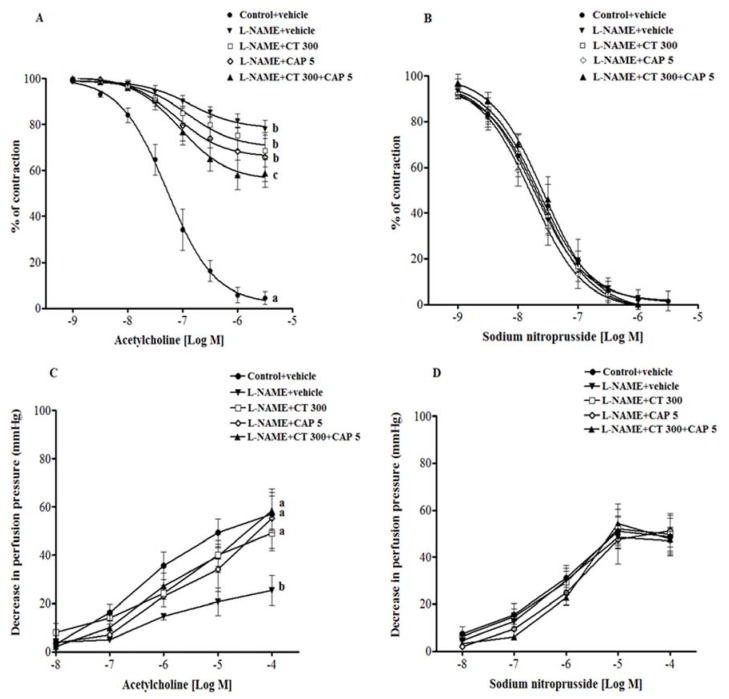
Vascular responses to acetylcholine ((**A**) in aortic rings; (**C**) in mesenteric vascular beds), and sodium nitroprusside ((**B**) in aortic rings; (**D**) in mesenteric vascular beds) in tissue samples collected from Control + vehicle, l-NAME + vehicle, l-NAME + CT 300, l-NAME + CAP 5 and l-NAME + CT 300 + CAP 5 groups. Values are mean ± SEM (*n* = 6 for each group). Data bearing different letters were significantly different (*p* < 0.05).

Endothelium-dependent vasorelaxation responses to Ach (0.01 µM–3 µM) were significantly reduced in aortic rings from l-NAME hypertensive rats compared to control rats (3 µM Ach, 77.9 ± 2.7 *vs.* 4.5% ± 4% of contraction) (*p* < 0.001). Neither CT extract nor captopril supplementation improved vascular response to Ach. However, an improvement of Ach-induced vasorelaxation was observed in aortic rings collected from CT extract plus captopril group compared to untreated group (3 µM Ach, 58.7% ± 6% of contraction; *p* < 0.05) (Figure 2A). In addition, vasorelaxation response to SNP, an NO donor, did not differ significantly across experimental groups (Figure 2B).

Vasorelaxation response to Ach (0.1 µM–0.1 mM) in the mesenteric vascular bed was significantly blunted in l-NAME preparations compared to the response to control preparations (0.1 µM Ach, 25.4 ± 6.1 *vs.* 57 ± 7.5 mmHg) (*p* < 0.01). Treatment with CT extract, captopril, and CT plus captopril caused an improvement in the response to Ach compared to untreated preparations (0.1 µM Ach, 49.2 ± 7.3, 55.2 ± 12.3 and 58.5 ± 7.6 mmHg, respectively) (*p* < 0.05; Figure 2C). However, there was no significant difference in the vasorelaxation responses to SNP amongst the groups, indicating normal vascular smooth muscle cell function (Figure 2D).

### 3.4. Effects of CT Extract and Captopril Supplementation on Oxidative Stress Status in l-NAME Hypertensive Rats

In CT extract treated-hypertensive group, the elevation of O_2_^−^ production and plasma MDA was significantly reduced (*p* < 0.05). Moreover, captopril treatment or combination of CT extract and captopril treatment restored oxidative stress markers to the levels of normal rats (*p* < 0.05) (Table 2).

### 3.5. Effects of CT Extract and Captopril Supplementation on Plasma Ang II and Plasma NOx Levels

**Figure 3:**
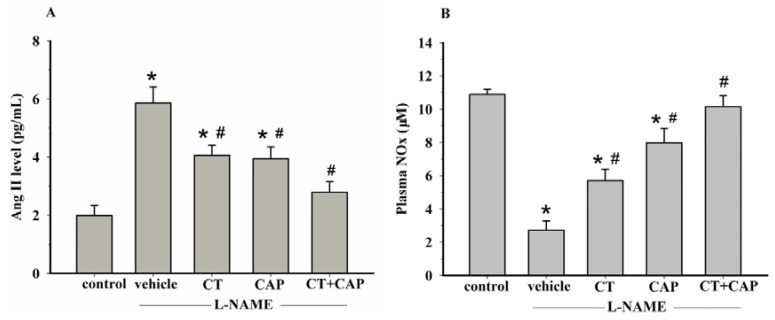
Effect of CT extract and captopril on plasma Ang II (**A**) and plasma NOx (**B**) levels in control, vehicle, CT, CAP and CT + CAP groups. Data are expressed as mean ± SEM (*n* = 5 in each group). * *p* < 0.05 *vs.* control group, ^#^ *p* < 0.05 *vs.* vehicle group. Control = normal control rats, vehicle = l-NAME hypertensive rats received vehicle, CT = l-NAME hypertensive rat that received CT (300 mg/kg/day), CAP = l-NAME hypertensive rats that received captopril (5 mg/kg/day) and CT + CAP = l-NAME hypertensive rats that received CT (300 mg/kg/day) together with captopril (5 mg/kg/day).

An increase in plasma Ang II was found in l-NAME hypertensive rats compared to the level in control rats (5.9 ± 0.6 *vs.* 2 ± 0.3 pg/mL). Supplementation with CT extract or captopril alone partially reduced plasma Ang II level compared to untreated rats (4.1 ± 0.3 and 3.9 ± 0.4 pg/mL, respectively). Furthermore, treatment with CT extract plus captopril normalized the plasma Ang II level in hypertensive rats more than CT or captropril alone (2.8 ± 0.4 pg/mL) (*p* < 0.05) (Figure 3A). Plasma nitrate/nitrite level was decreased in l-NAME treated rats comparing to control rats (2.72 ± 0.56 *vs.* 10.88 ± 0.31 μM, respectively) (*p* < 0.05). Hypertensive rats that received CT extract (300 mg/kg/day) or captopril (5 mg/kg/day) showed a significant increase in plasma nitric oxide metabolites (5.71 ± 0.67 and 7.97 ± 0.87 μM, respectively) (*p* < 0.05). Captopril (5 mg/kg/day) plus CT extract (300 mg/kg/day) supplementation restored plasma nitric oxide metabolites in l-NAME hypertensive rats (10.14 ± 0.665 μM) (Figure 3B) (*p* < 0.05).

### 3.6. Effects of CT Extract and Captopril Supplementation on Aortic eNOS and AT_1_R Protein Expression in l-NAME Hypertensive Rats

**Figure 4:**
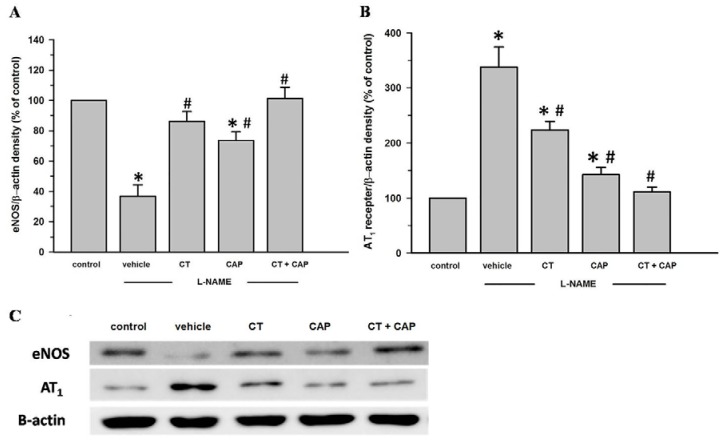
Aortic protein expression of endothelial nitric oxide syntase (eNOS) (**A**) and Angiotensin type 1 receptor (AT_1_R) (**B**) in the thoracic aorta of control, vehicle, CT, CAP and CT + CAP groups; Data are expressed as mean ± SEM (*n* = 5 in each group); Bottom: Representative bands of eNOS and AT_1_R (**C**). Control = normal control rats, vehicle = l-NAME hypertensive rats that received vehicle, CT = l-NAME hypertensive rat that received CT (300 mg/kg/day), CAP = l-NAME hypertensive rats that received captopril (5 mg/kg/day) and CT + CAP = l-NAME hypertensive rats that received CT (300 mg/kg/day) together with captopril (5 mg/kg/day). Data are expressed as a percentage of the values determined in the control group from the same gel. * *p* < 0.05 *vs.* control group, ^#^ *p* < 0.05 *vs.* vehicle group.

Downregulation of eNOS protein expression in aortic tissues was observed in l-NAME-induced hypertensive rats (*p* < 0.05). CT extract alone partially increased the expression of eNOS protein level in aortic tissues (*p* < 0.05) (Figure 4A). Interestingly, treatment with captopril or CT extract plus captopril completely restored eNOS protein expression in aortic tissues (*p* < 0.05). In addition, upregulation of AT_1_R expression in aortic tissues was also observed in the l-NAME treated rats (*p* < 0.05). CT extract or captopril partially suppressed l-NAME induced-AT_1_R overexpression in aortic tissues (*p* < 0.05). Interestingly, treatment with CT extract plus captopril completely reversed AT_1_R expression in aortic tissues of hypertensive rats (*p* < 0.05) (Figure 4B).

## 4. Discussion

Similar to previous studies, the present study shows that l-NAME caused hypertension with high vascular resistance. l-NAME also induced other abnormalities in rats, including endothelial dysfunction, increased vascular O_2_^−^ production, high plasma MDA, increased plasma Ang II andNOx levels, upregulation of AT_1_R protein expression and downregulation of eNOS protein expression. CT extract alleviated hemodynamic alterations, endothelial dysfunction, increased oxidative stress markers, NO inactivation and RAS activation. Both CT extract and captopril partially reversed the abnormalities in l-NAME hypertensive rats. Furthermore, CT extract enhanced antihypertensive effects of captopril in l-NAME hypertensive rats. A combination of CT extract and captopril treatment produced greater antihypertensive effects than either CT extract or captopril.

High blood pressure and high vascular resistance induced by chronic blockade NO synthesis using l-NAME were observed in this study. It is well documented that lack of NO constricts the vascular bed and causes high vascular resistance and high blood pressure [28]. Several studies have shown that endothelial dysfunction in l-NAME hypertension is associated with a decrease of NO level, downregulation of eNOS expression and increases in O_2_^−^ production and plasma MDA [29,30]. We confirmed that endothelial dysfunction observed in this study was not only caused by a reduction of NO synthesis but also involved oxidative stress. Increased O_2_^−^ production is known to decrease NO bioavailability, since it rapidly reacts with NO to produce ONOO^−^ [31]. CT extract reduced blood pressure and vascular resistance as well as endothelial dysfunction in hypertensive rats. Oxidative stress markers, vascular O_2_^−^ production and plasma MDA level were attenuated in hypertensive rats after CT extract treatment. Therefore, it is likely that the anti-oxidative stress properties are one mechanism by which CT extract reduces blood pressure in this animal model of hypertension. Salem and coworkers recently demonstrated phenolic composition and antioxidant properties of CT extract [32]. In addition, it has been reported that flavonoids were the main component of CT extract and strongly scavenge free radicals [33]. Therefore, the anti-oxidative effect of CT extract might play an important role in increasing NO bioavailability and improve endothelium-dependent vasodilation. This is supported by increased plasma NOx and upregulated of eNOS expression in l-NAME hypertension with CT extract supplementation.

Similar observations have been made in hypertensive rats treated with captopril (5 mg/kg). Captopril is mostly used as an antihypertensive drug. It is known that elevation of blood pressure in NO deficient hypertensive rats is associated with the activation of RAS. Our findings here are in accordance with several previous studies. For example, Zanchi and coworkers [11] found that there was an increase in plasma renin activity in rats with chronic NO synthase inhibition. A reduction of papillary blood flow induced by chronic inhibition of NO synthesis was partially mediated by the effects of Ang II, via stimulation of AT_1_R [34]. In addition to its effects in inhibiting ACE, captopril has been reported to have antioxidant activity, since it is composed of thiol groups [16]. Captopril is known to enhance eNOS protein expression in the descending aorta of rats with l-NAME-induced hypertension [5]. Thus, the antihypertensive effect of captopril in l-NAME hypertensive rats may involve at least two mechanisms, namely its ACE inhibitor and antioxidant properties. In subsequent studies in l-NAME treated rats, treatment with CT extract plus captopril was more effective in improving hemodynamic alterations, endothelial dysfunction, plasma NOx, eNOS expression and oxidative stress than either captopril or CT extract alone. These results suggest that CT extract and captopril have a synergistic effect on reducing blood pressure in l-NAME hypertension, and that this effect is associated with increased NO bioavailability and reduced oxidative stress. These findings are consistent with the recent study that combined treatment with resveratrol and captopril alleviated aortic remodeling and fibrosis in renovascular hypertensive rats via its antioxidant action and nitric oxide generation [35].

Activation of the RAS has been reported to contribute to the development of hypertension in the l-NAME-induced hypertension model. The current study showed that there were increases in plasma Ang II levels and AT_1_R protein expression in l-NAME hypertensive rats. This is consistent with a previous study that reported a high level of plasma Ang II in l-NAME treated spontaneous hypertensive rats [36]. The authors suggested that there were increases in Ang II synthesis and/or secretion. Furthermore, the levels of plasma renin activity, plasma Ang II, and renal renin mRNA were elevated following l-NAME treatment [37]. It was recently proposed that AT_1_R expression is upregulated in the renal cortex in l-NAME hypertensive rats [9]. The mechanism by which l-NAME-induced hypertension is associated with upregulation of AT_1_R expression is unknown, but it is possible that NF-κB directly upregulates AT_1_Rs. In human aortic smooth muscle cells (HASMC) exposed to oxidative stress, AT_1_R is upregulated in vascular tissue, and this was inhibited by an antioxidant or siRNA against p65 subunit of NF-κB [38]. In the current study, we found that the antihypertensive effect of CT extract was also related to the RAS, since both plasma Ang II and AT_1_R expression were reduced in l-NAME hypertension after treatment with CT extract. Other evidence also supports the idea that CT extract affects the RAS. Liu and coworkers found that safflower yellow reduced blood pressure in SHR associated with the decrease of plasma renin activity and Ang II level [22]. In our study, the attenuation of RAS by CT extract in l-NAME hypertensive rats might be mediated by CT extract’s antioxidant and ACE inhibitor properties. Similarly, captopril was able to decrease plasma Ang II and protein AT_1_R expression in the l-NAME treated group, possibly due to its ACE inhibitor activity. Moreover, in hypertensive rats, combination treatment with CT extract and captopril was more beneficial than treatment with either CT extract or captopril.

Since captopril may have some side effects [17], it would be desirable to reduce the prescribed dose for clinical treatment. Combined treatment captopril with CT extract would provide alternative treatments for hypertension. However, CT extract contains several beneficial compounds including quinochalones, flavonoids, alkaloids, polyacetylene, aromatic glucosides, and organic acids [19]. Further study is required to obtain the antihypertensive effect of the main component from CT extract *in vivo*.

## 5. Conclusions

These findings demonstrated that the development of hypertension in l-NAME treated rats was associated with endothelial dysfunction, decreased eNOS protein expression, NO bioavailability, oxidative stress and RAS activation. Combined therapy with CT extract and captopril was more effective than CT extract or captopril alone since this combined therapy normalized hemodynamic alterations, RAS, NO bioavailability, oxidative stress markers and improved endothelial dysfunction in NO-deficient hypertension.

## Figures and Tables

**Table 1:** Effects of CT extract and captopril supplementation on hemodynamic status.

Parameters	Control + Vehicle (*n* = 12)	Control + CT 300 (mg/kg/Day) (*n* = 8)	l-NAME + Vehicle (*n* = 12)	l-NAME + CT 75 (mg/kg/Day) (*n* = 8)	l-NAME + CT 150 (mg/kg/Day) (*n* = 8)	l-NAME + CT 300 (mg/kg/Day) (*n* = 12)	l-NAME + CT 500 (mg/kg/Day) (*n* = 8 )	l-NAME + CAP 5 (mg/kg/Day) (*n* = 12)	l-NAME + CAP 5 + CT 300 (mg/kg/Day) (*n* = 12)
SP (mmHg)	120.7 ± 1.8 ^a^	119.8 ± 1.7 ^a^	199.3 ± 3.4 ^b^	186.7 ± 5.7 ^c^	178.6 ± 3.7 ^c^	163.3 ± 3.2 ^d^	169.5 ± 2.4 ^c,d^	151.8 ± 3.7 ^e^	125.3 ± 2.6 ^a^
DP (mmHg)	77.3 ± 3.3 ^a^	78.6 ± 1.8 ^a^	140.3 ± 4.1 ^b^	124.28 ± 7.00 ^c^	109 ± 4.9 ^d^	106.9 ± 2.2 ^d^	118.46 ± 3 ^c,d^	87.8 ± 4.6 ^a^	75.8 ± 3.6 ^a^
MAP (mmHg)	91.7 ± 2.6 ^a^	92.3 ± 1.5 ^a^	160 ± 3.8 ^b^	145.11 ± 6.36 ^c^	132.2 ± 4.1 ^d^	125.7 ± 2.4 ^d,e^	135.5 ± 2.7 ^c,d,e^	109.1 ± 3.8 ^b,c,d,e,f^	92.3 ± 2.8 ^a^
PP (mmHg)	44.9 ± 3.1 ^a,d^	40.4 ± 1.9 ^a^	59 ± 1.6 ^b^	59 ± 2.4 ^b,c^	63.3 ± 1.6 ^b^	54.6 ± 1.8 ^b,c^	50.9 ± 1.5 ^b,c,d^	60 ± 2.1 ^b^	50.3 ± 3.3 ^c,d^
HR (beats/min)	362.9 ± 3.7 ^a,b^	341.5 ± 12.1 ^a^	399 ± 8.1 ^a,b^	362.8 ± 10 ^a,b^	356.8 ± 9.2 ^a,b^	359.9 ± 9.9 ^a,b^	379 ± 4.9 ^a,b^	365.6 ± 14.5 ^a,b^	349.6 ± 10.1 ^a^
HBF (mL/min/100 g tissue)	6.3 ± 0.3 ^a^	5.9 ± 0.2 ^b^	3.1 ± 0.2 ^b^	3.6 ± 0.3 ^b^	3.9 ± 0.2 ^b^	4.7 ± 0.2 ^c^	4.4 ± 0.2 ^c^	4.7 ± 0.2 ^c^	6.2 ± 0.5 ^a^
HVR (mmHg/min/100 g tissue/mL)	13.6 ± 0.9 ^a^	15.7 ± 0.9 ^a^	44.2 ± 2.4 ^b^	44.3 ± 6.9 ^b^	34.6 ± 1.9 ^c^	26.8 ± 1 ^d^	28. 4 ± 1.2 ^d^	23.7 ± 1.3 ^d^	15.6 ± 1 ^a^

Data are expressed as mean ± SEM. SP: systolic blood pressure, DP: diastolic blood pressure, MAP: mean arterial pressure, PP: pulse pressure, HR: heart rate, HBF: hindlimb blood flow, HVR: hindlimb vascular resistance. Data bearing different letters were significantly different (*p* < 0.05).

**Table 2:** Effects of CT extract or captopril supplementation on oxidative stress status.

Parameters	Control + Vehicle (*n* = 8)	l-NAME + Vehicle (*n* = 8)	l-NAME + CT 300 (mg/kg/Day) (*n* = 8)	l-NAME + CAP 5 (mg/kg/Day) (*n* = 8)	l-NAME + CAP 5 + CT 300 (mg/kg/Day) (*n* = 8)
**Luciginine-Enhanced Chemiluminescence (count/mg dry wt/min)**	46.8 ± 4.4	116 ± 8.4 *	94.3 ± 9.8 *^,#^	52.9 ± 2.4 ^#^	53.9 ± 4.6 ^#^
**MDA (µM)**	4 ± 0.5	9.8 ± 0.5 *	5.4 ± 0.4 *^,#^	4.8 ± 0.2 ^#^	4.3 ± 0.3 ^#^

MDA: malondialdehyde. Values are mean ± SEM. *p <* 0.05 * *vs.* control group, *p <* 0.05 ^#^ *vs*. l-NAME + vehicle group.
